# Lived experiences of caregivers of children with autism spectrum disorder in Kenya

**DOI:** 10.4102/ajod.v8i0.435

**Published:** 2019-04-25

**Authors:** Lizahn G. Cloete, Evans O. Obaigwa

**Affiliations:** 1 Department of Interdisciplinary Health Sciences, Stellenbosch University, Stellenbosch, South Africa

## Abstract

**Background:**

Autism spectrum disorder (ASD) is a global public health concern. In African countries such as Kenya, there is a greater need for establishing support services for developmental disorders such as ASD. The emotional, social and economic burden of ASD on caregivers is unknown because of a number of challenges. Citizens of Kenya have a unique view of disability and inclusion.

**Objectives:**

To explore the perspectives of caregivers who are responsible for caring for both family and children living with ASD and to highlight the needs of children with ASD as well as the needs of their caregivers.

**Method:**

A qualitative, descriptive phenomenological study utilising focus group discussions (FGDs) was conducted. Verbatim transcription was used. QSR N ’Vivo 10 was used to organise and analyse the data. Content analysis was used to identify important ideas and concepts.

**Results:**

One theme, namely ‘the burden of caring for children with ASD’, was identified. Children with ASD and their caregivers experience isolation and stigmatisation.

**Conclusion:**

Occupational therapists in Kenya should collaborate with the relevant national and global stakeholders for the promotion of the inclusion of children with ASD and their families. Responsive and context-appropriate occupational therapy interventions may begin to address service barriers.

**Keywords:**

autism spectrum disorder; children; needs of caregivers; context-appropriate services.

## Introduction

Autism spectrum disorder (ASD) is a disorder associated with individuals with autism, Asperger’s disorder or a developmental disorder not otherwise specified (American Psychiatric Association 2013; World Health Organization 2013a). The onset of this neurodevelopmental disorder occurs in childhood, and it can persist into adulthood. Characteristic symptoms include impaired social communication and interaction, as well as restricted, stereotyped behaviour, activities and interests (American Psychiatric Association 2013; World Health Organization 2013a). Since the discovery of ASD, it has been difficult to establish its cause; however, studies have attempted to connect the aetiology of ASD with heredity (Betancur 2011), environmental factors (Landrigan 2010), advanced age in both parents (Ronald & Hoekstra 2011) and, most disputed of all, vaccines (Gona 2016). However, alternative paradigms such as neurodiversity view ASD as a variation in the neurology of individuals and part of the identity of such individuals (Jaarsma 2012).

The discrepancies in ascertaining the exact information on the causes of ASD, especially among Africans, hamper the efforts required to address the ASD-related needs and service provision of Kenyans. Consequently, individuals with ASD and their families in Kenya have poor quality of life. In 2008, the member states of the United Nations (UN) emphasised the need to take ASD seriously as a public health concern, and in 2012, the UN General Assembly agreed to adopt measures to address the socio-economic needs of children with ASD, family members and society. This initiative has been difficult to implement in Africa because of the lack of resources, poor advocacy, inadequate collaborative efforts and ineffective strategies (World Health Organization 2013b). In Kenya, as in other developing African nations, developmental disorders such as ASD have attracted little attention from the government and other stakeholders regarding establishing support services for individuals with ASD (Njogu 2009; Republic of Kenya 2009). Bayat (2014) suggests that there is a general lack of literature on the needs of African children with disabilities or the views of Africans towards disabilities. Service provision and resource allocation in English-speaking developed countries is also much more advanced than those in the Global South. These discrepancies have hampered efforts to address ASD-related needs and service provision in countries like Kenya (Njogu 2009; Republic of Kenya 2009). There is also a need to develop advanced diagnostic and reporting systems before meaningful data on the rising prevalence of ASD (American Psychiatric Association 2013; Matson & Kozlowski 2011) in the region could be captured. To ensure the development of appropriate networks for service delivery to children with ASD, more information is required on the needs as expressed by caregivers of children with ASD. This study therefore posed the following research question: what are the lived everyday experiences of the caregivers of children with ASD at Kenyatta National Hospital?

Kenya is a country of several tribes, who have different cultural beliefs and practices. Cultural and spiritual beliefs strongly influence the health-related experiences of people in Kenya. Similarly, cultural and spiritual beliefs often inform the beliefs and attitudes of the caregivers and community members in a manner that negatively influences caregiving practices of children with ASD. Furthermore, limited knowledge about the aetiology of ASD may restrict the type and nature of research data that could be collected, which, in turn, may hamper the development of and access to support services. The Kenyan government and other stakeholders have not yet taken initiatives regarding establishing prevalence, the impact of ASD, and allocating resources for the research and training of professionals to support children with ASD and their families. According to Kenya National Bureau Statistics (2015), Kenya has a population of about 40 million, and the United Nations Children’s Fund (UNICEF) estimates that 1% of children in Kenya live with disabilities, including those with ASD, which translates to about 400 000 children with disabilities in Kenya. The Kenya Occupational Therapists Association (KOTA) (n.d.) indicates that there are 900 occupational therapists (OTs), which is below the WHO threshold of about 750 occupational therapists per a population of 1 million people (WHO Rehabilitation 2030 2017). The number of OTs in Kenya is therefore not enough to address the needs of children with ASD and their caregivers. Considering the challenges associated with health care provision of children with disabilities, the institutions responsible for providing such care are unprepared at best (Seif et al. 2008). Within the Kenyan health system, OTs, as crucial members of the multi-disciplinary team, may contribute to the care, integration and inclusion of children with ASD. A holistic approach to address the needs of children with ASD would include the provision of information pertaining to ASD through caregiver education, research and publication. In addition, advocacy for rights of children to play, socially participate and engage in family routines, independent living and the right to enter into gainful employment (Kuhaneck & Watling 2015) later in life are equally important. Rehabilitation interventions should focus on neurodevelopmental and sensory-motor approaches within occupational therapy rehabilitation to address the characteristic symptoms experienced by children with ASD. Interventions that focus on meeting developmental milestones may enhance sensory processing and sensory-motor performance towards enabling self-care and participation in play. With adolescents, occupational therapy rehabilitation may focus on social behavioural performance, transition to work and independence in the community (Case-Smith & Arbesman 2008). A combination of the above-mentioned approaches may effectively mobilise resources for inclusion in all areas of life.

Caregivers of children with ASD in Africa have a variety of experiences, some of which are different from caregivers who live in countries with more resources like the USA, UK and Northern Ireland (World Health Organization 2013a). In a study conducted in the USA, Brien (2007) suggests that a diagnosis of autism, as well as the severity of the impairments, can act as external stressors to the caregivers and family. Africans mostly view persons with disabilities negatively; for instance, many Kenyans believe that illness and disability are the result of ancestral dissatisfaction (Gona 2016). The inclusion of children with disability as equal members of society may therefore not be a priority. This view of disability is in contrast with the Ubuntu philosophy. Jolly (2011) describes Ubuntu as the essence of being human. Within the Ubuntu philosophy, the boundaries between nuclear and extended families are enmeshed. Being part of the community offers benefits and requires equal responsibility where each person acknowledges his or her own connectedness with and interdependence on the other. The view of disability as misfortune excludes individuals with disabilities from the benefits of Ubuntu that prescribes collective ownership of success and misfortune (Wilson & Williams 2013). In her ethnographic work conducted with students labelled as being on the autism spectrum, Baines (2014) challenged readers to see beyond disability labels by focusing on the unique talents, beliefs and dreams of the individual.

Jaarsma (2012) argues that to accept these individuals as a special group with specific needs, there should be a reduction of societal misconceptions towards ASD. Other studies conducted in Iran, Tanzania and Poland have reported that there are also gender differences in terms of how the caregivers experience the impact of taking care of a child with ASD. A child with ASD presents many emotional, social and economic challenges to the family (Bilgin & Kucuk 2010; Diken 2006; Gona 2016; Landrigan 2010). Caregivers spend a lot of energy balancing the needs of the family and those of the children living with ASD (Ghanizadeh & Alishahi 2009). In addition, Ambikile and Outwater (2012) reported that mothers as caregivers of children with ASD have poorer mental health than the general population. Mak and Kwok (2010) conducted a study in Hong Kong and reported that there is ambiguity concerning whether the diagnosis of ASD can contribute to caregivers’ stress perceptions. People experiencing ambiguous loss have difficulties in making decisions regarding the care of children with pervasive developmental disorders (Ambikile & Outwater 2012; Brien 2007). Mothers generally experience more stress symptoms than fathers do, particularly when they learn about their child’s health condition; and uncertainty regarding how to deal with their child’s behaviours was one of many concerns (Ambikile & Outwater 2012; Brien 2007; Dabrowska & Pisula 2010; Ghanizadeh & Alishahi 2009).

Based on the findings of the UN General Assembly resolution report, there is a need for the empowerment of health care professionals with adequate knowledge pertaining to the management of ASD, caregiver education and other collaborative interventions. Similar qualitative studies confirmed that the behaviours associated with ASD affect those involved in the care of such children, and that support (including training programmes) from family, government and other organisations helps families deal with these daily challenges (Bilgin & Kucuk 2010; Gona 2016; Luther, Canham & Cureton 2005; Republic of Kenya 2014; Samadi & McConkey 2011; World Health Organization 2013b).

In the USA, Plumb (2011) explored how having a child with ASD influences a couple’s lives, and established that despite the extreme demands of caring for such a child, the parents usually achieve resilience and that the child’s condition may even bring the couple together and also that participants’ marital relationships generally survived because of social support as well as professional help. Starr and Foy (2010) also found that Canadian parents perceived their options in terms of general educational issues, and the teacher support provided to their children, as adequate. Teachers’ knowledge in managing children with ASD behaviours reassured parents that their children were receiving adequate support at school. Other studies have shown that these children need long-term care, which interferes with families’ quality of life, in respect of the costs of service provision and special education, and the loss of family income (Republic of Kenya 2009; Ruiz-Robledillo et al. 2014; Starr & Foy 2010; Siman-Tov & Kaniel 2011; World Health Organization 2013b). In turn, the caregivers of African children with disabilities may be faced with different challenges such as food insecurity (and malnutrition), poverty, political unrest and general beliefs around the causes of the disability. Due to these factors, families may experience unique barriers that may keep them from contributing towards the implementation of a care model that could address the social determinants of health as well as the issues related to caring for the child and his or her caregivers.

Another study in the USA compared two groups of siblings – that is, those with ASD and their siblings with no disability. This study found that various factors strengthen relationships between family members, or between the child’s parents. Rivers and Stoneman (2003) examined similar relationships and recommended family support in such families. In most African countries, children with disabilities are often invisible, mistreated by caregivers and have poor access to health care (Bayat 2014). Unlike caregivers of children without ASD, those of children with ASD were utilising health services such as speech therapy, occupational therapy and other special interventions – which were expensive, unavailable and require trained professionals to implement (Republic of Kenya 2009; Ruiz-Robledillo et al. 2014; Starr & Foy 2010; Siman-Tov & Kaniel 2011; World Health Organization 2013b). Although the health care service needs of children with ASD may be similar across the world, the availability of resources and the nature of the resources needed by African children may differ starkly from agreed service structures. Therefore, the aim of this study sets out to explore how an understanding of the lived experiences of caregivers of children with ASD could inform the development of appropriate services in Kenya.

## Research methods and design

### Study design

Cognisant of the emerging theoretical framework of critical autism studies, which combines medical and social approaches to explore disability (Davidson & Orsini 2013), this study used a qualitative, descriptive, phenomenological study (Carpenter & Suto 2008; Ritchie et al. 2013). Participants engaged in four focus group discussions (FGDs) for data collection. Phenomenology as a qualitative methodology enabled the participants to share their experiences and voice their concerns (Liamputtong 2007). The phenomenological approach entails finding the meaning in human experiences, as related by the participants in the situation (Carpenter & Suto 2008; Ritchie et al. 2013). The process allowed the researcher to understand the perspectives of study participants, as recounted by the participants themselves. A phenomenological approach is also suitable for understanding subjective experiences, and understanding what motivates people to behave in a certain manner (Carpenter & Suto 2008; Ritchie et al. 2013). The participants’ stories yielded rich information in Kiswahili (the official language spoken by over 90% of the population in Kenya).

### Setting

The study took place at the occupational therapy clinic, one of the 22 outpatient clinics of Kenyatta National Hospital.

### Study population and sampling strategy

The study population consisted of the caregivers of 300 children with ASD who were attending the occupational therapy clinic at the hospital during the period of study between June and July 2015. Twenty-four participants were selected with purposeful sampling. Although mainly primary caregivers were selected to participate, one mother-in-law, who had taken care of her grandchild with ASD before transferring her caregiving role to the biological parents, was selected. Her rich accounts of the experience of caring for the child of her daughter and son-in-law contributed to answer the research question in a manner that resonated with caregiving approaches of taking collective responsibility in African cultures.

Each of the four focus group sessions comprised six caregivers, taking into consideration their different socio-economic backgrounds, cultures and age groups (Carpenter & Suto 2008; Ritchie et al. 2013). Participants who were related to each other (e.g. mother and son-in-law) were allocated to different groups. The severity of the child’s condition was not considered during allocation. The inclusion criteria for the study were as follows: participants had to have been an adult, primary caregiver of a child with ASD who attended the hospital for the last 3 months before the research commenced and were willing to give informed consent.

### Data collection

The four focus group sessions each lasted about 90 min and were conducted in a neutral place every month (Carpenter & Suto 2008; Ritchie et al. 2013), especially structured to ensure confidentiality. Predetermined short, open-ended and non-threatening questions (Carpenter & Suto 2008; Ritchie et al. 2013) elicited responses that reflected participants’ beliefs as well as experiences of raising children with ASD (Onwuegbuzie et al. 2009; Ritchie et al. 2013).

Other questions probed for the accessibility of supportive services for these children and their families (Onwuegbuzie et al. 2009; Ritchie et al. 2013) (Appendix 1). Bracketing allowed the researcher throughout the research process to distinguish between practitioner and researcher roles. Hand-written notes were transcribed immediately after every focus group discussion (taking approximately 8 h) into clearly typed wording, including all observed non-verbal expression from the participants. Individual member-checking sessions of 20 min with all participants confirmed that the captured information reflected the true views of the participants. Data collection took 22 h in total. Data collection stopped after data saturation was reached (the recurrence of similar information in data sets). Video recording enhanced the credibility of the content shared by participants during the discussion.

Strategies used to obtain the approval of the participants for recording the session included establishing rapport with the participants and assuring them of complete confidentiality (Onwuegbuzie et al. 2009; Ritchie et al. 2013). Informed consent was obtained for taking notes as well as for recording the sessions.

### Data analysis

Inductive content analysis was performed (Carpenter & Suto 2008; Ritchie et al. 2013). The data were transcribed verbatim before analysis was done. To ensure that the transcription reflected the totality of the discussions and facilitated analysis, transcription conventions such as the use of symbols and word emphasis were used. To ensure that the transcribed data were accurate, the recorded discussions were watched and listened to, and cross-checked with the transcripts. During cross-checking, any non-verbal behaviour captured in the recording was added to the field notes.

The researcher read the transcribed data to gain familiarity with the text. The focus group question guide (Appendix 1) was instrumental in enabling the researcher to organise and summarise the data. Ideas and concepts were identified. The N’Vivo software package was used to organise and analyse the field notes and video files derived from the focus group discussion sessions (Ritchie et al. 2013). Meaningful units were coded. Codes were clustered into subcategories, which were then grouped into categories. The categories and subcategories were labelled with descriptive phrases (Table 1). The researcher noted important threads and ideas that recurred. Paradoxes and conflicting ideas were also explored. Similar opinions were merged (Onwuegbuzie et al. 2009; Ritchie et al. 2013). To ensure the rigour of the process, a qualitative researcher not involved in the research was consulted to create an audit trail (Carpenter & Suto 2008; Ritchie et al. 2013). Finally, the researcher interpreted the findings. Connections between subcategories and categories were established and reduced into one theme.

**TABLE 1:** The burden of caring for children. Theme, category, subcategories, and codes.

Category 1	The caregivers’ emotional dilemmas		
Subcategories	The society’s view towards ASD	Strained couple relationship	Coping with the situation
Codes	‘The society had disturbing views regarding ASD, viewing it as a result of God’s punishment’.	‘Immediately the doctor told us the child’s behaviour is because of a condition called ASD, he reminded me! I told you, don’t name child after your deceased father!’.	‘But I was in shock, I didn’t understand, I had never heard of it’.
	‘This one (meaning ASD) must be as a result of evil eye, consult the witchdoctor, they always tells me’.	‘I have seen husbands run away from their family because of this condition’.	‘I looked at the paediatrician in disbelief, walked out of his clinic and I thought my God why?’.
	‘I have been told all types of causes of this condition, he has been bewitched, they even told me to appease the gods and some even suggested I throw him away…’.	‘My husband is unfaithful these days, I do not know; I always ask myself is it because of our child’s condition? He has turned to be a drunkard, comes home late!’.	‘But I didn’t expect it can happen to me’.
	‘The mother must have been unfaithful’.	‘Our social life has been affected with my husband; even sex life is not what used to be, we don’t enjoy [*ourselves*] anymore because all of your energy is the child.’.	‘First it was denial, to accept that your child has a problem (shaking his head) it takes courage, time, and you tell yourself it’s ok I will wait’.
	‘Yes, you can see we do not marry [*into*] their family. These are the consequences.’.	‘when I saw them I was overwhelmed by joy, you know I stayed with him (grandson) he was difficult, he could break everything in the house, it’s when I asked whether my daughter’s marriage can last, its 3 years since, they are together because most of the husbands run away.’.	‘Acceptance took time once you accept you take care of the child’.
	‘These behaviours indicate possession by evil spirits, I know children who behave this way because of her.’ (Mother of a child with ASD).		‘It took a lot of spending and consultations to accept’.
			‘However, [*I*] am happy with colleagues they are very supportive, even my relatives.’.
			‘Yes, his (child) condition has made us (caregivers) advocate for them’.
			‘Sometimes you think “I’m alone” but, at the clinic you find those who are even worse but their caregivers are OK …’.
			‘I had mixed feelings, my husband took care of everything, he has been encouraging me’.
			‘Another thing is talking about his condition … those who listen I explain to them about ASD, to enlighten them and I also leave it to God’.

ASD, autism spectrum disorder.

### Ethical considerations

The Kenyatta National Hospital – University of Nairobi ERC – as well as the University of Stellenbosch Health Research Ethics Committee (S14/10/260) approved the study. All the principles of ethics in research were observed during the study, including provision of information concerning the study, and obtaining consent in a language well understood by caregivers before and during focus group discussions with caregivers (Onwuegbuzie et al. 2009; Ritchie et al. 2013). The researchers used pseudonyms to protect the privacy of all participants. The researcher ensured the study information was accurate by safeguarding the rigour and trustworthiness of the study through the inclusion of credibility, transferability, dependability, confirmability, triangulation of data, peer debriefing and member checking (Krefting 1991; Onwuegbuzie et al. 2009; Ritchie et al. 2013).

## Results

The main theme identified was ‘the burden of caring for children with ASD’. Although two categories were identified in the analysis of the findings, namely ‘the caregivers’ emotional dilemmas’ and ‘difficulty accessing crucial services in the management of children with ASD’, this article only reports on Category 1 and the three subcategories as displayed in Table 1.

### Theme: The burden of caring for children with autism spectrum disorder

This is the main theme to emerge, describing the caregivers’ daily challenges in taking care of their children with ASD. The caregivers highlighted their challenges, ranging from emotional predicaments to difficulties in accessing crucial services in the management of their children with ASD. For example, some caregivers said:

‘…he can be chaotic; you have no peace.’ (Drusila, a 70-year-old farmer and grandmother of a child with ASD)

‘…all your energy and attention is on this child.’ (Jane, a 37-year-old revenue collection officer and mother of a 4-year-old child with ASD)

‘…little support from the government; this will drain you out.’ (David, a 40-year-old refugee and father of a child with ASD)

In the category of ‘*caregivers’ emotional dilemmas*’, caregivers described the ordeals they undergo in their daily lives with their children with ASD. These range from being psychologically affected as an individual and as a family to the effects of the community’s perception.

#### The society’s view towards autism spectrum disorder

According to the perceptions of the caregivers, the community’s view of the causes of ASD is that they lie solely in three aspects: the woman (as the main cause), marrying into the wrong tribe and supernatural causes. Jane and Teresa reported that society believes that the causes of ASD are linked to the family of the woman, probably ‘as a curse to certain families on the woman’s side’ (Jane, a 37-year-old revenue collection officer and mother of a 4-year-old child with ASD).

Annete said that her husband blamed her for their child being diagnosed with ASD, for naming the child after her deceased father. She said,

‘I named this child after my father; so when we discovered he (the child) had this problem (ASD), my husband chased me out of our marital home – he insisted they don’t have such children in their blood.’ (Annete, a 26-year-old business lady and mother of a 5-year old child with ASD)

Therefore, it is not surprising that once the child is born with ASD, the man and the community blame the woman as the main cause, or actions linked to the woman. As Zipporah said: ‘The mother must have been unfaithful’ (Zipporah, a 28-year-old teacher and mother of a child with ASD, also Drusila’s daughter).

Jane also said: ‘These behaviours indicate possession by evil spirits. I know children who behave this way, because of her [the mother of a child with ASD]’ (Jane, a 37-year-old revenue collection officer and mother of a 4-year-old child with ASD).

On the other hand, there are erroneous beliefs about the causes of ASD as ‘punishment from the gods’ (John and Linda), and therefore, the solution is either to do away with the child with ASD or to perform a ritual ‘to appease the gods’ and safeguard the child. Kezie, Zipporah, John and Linda suggested that a lack of information regarding the causes of ASD leads to some participants believing that some kind of ‘spell’ may have befallen the child, and that the solution required was ‘to appease the gods’. Jane and Ann indicated that society felt such children should not be living with their mother’s family. Jane reported what the community thought about her child’s condition: ‘She [*the child with ASD*] is not ours now; the other grandparents can take her – that’s where she belongs’ (Jane, a 37-year-old revenue collection officer and mother of a 4-year-old child with ASD).

#### Strained couple relationships

The demands and behavioural implications of ASD wear families down, particularly in terms of intimacy between married couples. Annete wondered if her child’s condition was the cause of her husband’s unfaithfulness:

‘My husband is unfaithful these days; I don’t know; I always ask myself: is it because of our child’s condition? He has turned to being a drunkard … comes home late!’. (Annete, a 26-year-old business lady and mother of a 5-year old child with ASD)

Jane added (supporting her hand with her left arm, looking down, with a sad face):

‘Our social life has been affected, with my husband. Even our sex life is not what it used to be, we don’t enjoy it anymore; because all of your energy is the child’. (Jane, a 37-year-old revenue collection officer and mother of a 4-year-old child with ASD)

Having a child with ASD is heart-breaking particularly for a woman, and the hurt is often extended to close family members. The difficult situation of managing the destructive behaviours of children with ASD often results in divorce. Drusila stated (with tears in her eyes, hands squeezing her handkerchief):

‘…when I saw them, I was overwhelmed by joy; you know, I stayed with him [*her grandson*], he was difficult, and he could break everything in the house. It is when I asked whether my daughter’s marriage could last; its three years since they are together. Because most of the husbands run away.’ (Drusilla, a 70-year-old farmer and grandmother of a child with ASD)

#### Coping with the situation

Learning to accept their children’s disabilities, receiving support and initiating the role of advocacy were important phases of learning necessary to cope with the reality of having a child with ASD. Different participants reacted differently on learning about their child’s condition: Lydia, Boaz and Kezie could not believe the diagnosis, and remained in *‘shock and denial’.* Teresa, on the other hand, had similar feelings to Aunt Ann: it was difficult for them to accept that their children had been accurately diagnosed.

James added: ‘First it was denial; to accept that your child has a problem… (Shaking his head): “…it takes courage, time – and you tell yourself, it’s okay, I will wait…”’ (James, a 30-year-old government employee and father of a child with ASD).

Support from both family and colleagues, particularly for those who were employed, played a crucial role in the caregivers’ adjustment to their children’s condition. Being employed reduced the burden of having to take care of children with ASD. For Irene (and other employed participants), colleagues could provide support and shield absences from work by enabling them to work in shifts. Linda noted that as much as managing children with ASD might be challenging, support from those who understand such children enabled caregivers to adjust. As she said: ‘I was blessed to have a good housemaid, who has taken care of my child all this time’ (Linda, a single mother of a 5 and half year-old child with ASD).

It is also important to note that attending therapy sessions contributed immensely to the adjustment of caregivers to their children’s condition. For instance, most of the participants indicated that meeting with other caregivers whose children had similar problems, or worse, played a vital role in accepting their children’s condition. Ann (and other participants) noted: ‘Sometimes you think, “I’m alone”; but at the clinic, you find those who are even worse, but their caregivers are okay…’ (Ann, a 24-year-old, had a high school education and was employed as a caregiver of a child with ASD).

Trizia also noted ‘…but I am happy with colleagues, they are very supportive, even my relatives’. Zipporah added: ‘I had mixed feelings; my husband took care of everything, and he has been encouraging me’ (Zipporah, a 28-year-old teacher and mother of a child with ASD, also Drusila’s daughter).

Interestingly, the study established that other caregivers had gone a step further – to initiate an advocacy role for these children. This was usually after going through a lot and realising that no association was willing to speak on their behalf. Grace said: ‘Yes, his [*her child’s*] condition has made us [*the caregivers*] advocate for them [*the children*]’ (Grace had a high school education and was employed as a caregiver of a child with ASD).

Kezie added: ‘Another thing is talking about his condition…those who can listen, I explain to them about ASD – to enlighten them; and I also leave everything to God’ (Kezie, mother of a 6-year-old child with ASD).

For these caregivers, the coping process was difficult; however, learning from other caregivers with similar challenges enabled them to adjust. Those who had support from family members were also able to adjust. However, there was a group of caregivers who realised there was discontentment in the community and took it upon themselves to educate and advocate for the well-being of their children with ASD. Though coping with their children’s condition was difficult, depending on different circumstances the caregivers were able to take care of their children with ASD.

## Discussion

The main finding of this study is that participants experienced an emotional burden when taking care of children with ASD. Participants initially found it difficult to accept and process the diagnosis. The communities in which the participants live believed that ASD is caused by women’s previous forbidden cultural actions or through marriage to members of certain prohibited tribes. Some participants blamed supernatural powers for causing ASD. These findings were consistent with the study conducted by Gona (2016), who also reported on the lack of information about the causes of ASD. Inadequate information on the causes of ASD may lead to people developing their own misconceptions, based on their spiritual and cultural beliefs. Critical questions regarding the value of Ubuntu as it applies to children with ASD and other disabilities may create opportunities for revisiting existing knowledge frames of caregivers, extended families and community members in general. Educating caregivers and community members on viewing children with ASD as individuals with unique identities and specific contributions to the family and the community may strengthen the neurodiversity perspective in African communities. A deeper exploration of how caring for a child with ASD informed or changed the caregivers, own cultural and spiritual beliefs of disability may contribute to addressing the stigma integrating children with ASD into African communities.

The challenges faced by participants included difficult behaviours in their children, which often resulted in isolation from family circles and strained marriage relations. Dabrowska and Pisula (2010) suggested that families taking care of children with ASD require more social and financial support to cope well with their children’s condition (Republic of Kenya 2014; Samadi & McConkey 2011). Social isolation as a method of protecting their children from the negative reactions of society may be addressed by educating and further exploring the support tribal councils could potentially provide within communities. This may enable tribal leaders to engage with the challenges and opportunities experienced by caregivers as contributing members of the community. Implementing the Ubuntu philosophy on caregiving practices of children with ASD may enable occupational therapy practitioners and caregivers to learn collectively how to plan and develop comprehensive support services for children and caregivers.

In their unfavourable circumstances, the caregivers taking care of children with ASD developed mechanisms to enable them to live a balanced life. Through bringing their children to the occupational therapy department for therapy, they learnt that other caregivers faced similar predicaments. Participants’ accounts of these interactions confirmed the importance of creating a space in which caregivers of children with ASD could interact informally before, during or after therapy sessions. The participants valued such peer support from other couples. This aspect should be seen as crucial in the management of children with ASD.

Because of the community’s spiritual and cultural beliefs that disability is an evil, caregivers of children with ASD are isolated and often did not receive the needed support from partners and close family members. The support from family members and colleagues was highlighted as an important element for caregivers’ adjustment, particularly for those caregivers who were working, as their colleagues were able to organise flexible working schedules to enable them to focus on the needs of their children. Similarly, Luong, Yoder and Canham (2009) stated that mothers cope well when they learn from those mothers whose children have similar problems. Social support plays a vital role in parents’ adjustment and in enhancing good health – a good strategy for therapists to utilise collaboratively with caregivers (Brien 2007; Ganz 2007; Gona et al. 2015; Luther, Canham & Cureton 2005; Ruiz-Robledillo et al. 2014; Samadi & McConkey 2011).

### Strengths and limitations

The views of the participants in the sample do not represent the views of all consumers of occupational therapy services. The findings do not reflect the views of carers of children with ASD who have never accessed occupational therapy services in the public health sector. A cross-sectional survey that reflects views of a wider population may be more representative of all caregivers of children with ASD in Kenya.

### Implications and recommendations

Rehabilitation care practitioners in Kenya could collaborate with caregivers to create awareness of the medical causes of ASD. Efforts should also focus on creating opportunities where caregivers, the community and service provided could engage on African perspectives of disability and how best to design services that complement the needs of caregivers and the broader community. Further research is required on exploring African perspectives as an additional view to neurobehavioural and neurodiversity perspectives of ASD. Moreover, a collaborative approach within a feminist theoretical framework may further illuminate the nature of support needed by male and female caregivers. Awareness campaigns and educational initiatives might also alleviate the public’s misconceptions and prejudices concerning this condition. A feminist perspective on the responsibility and role of women in the construction of health and wellness in African nations would illuminate the gaps in support provided to children with ASD and their caregivers. Furthermore, there is a need for multi-disciplinary collaboration across different sectors in order to streamline resources and train professionals in the utilisation of ASD care guidelines intended for dealing with spirituality and cultural beliefs related to ASD in relevant institutions.

## Conclusion

This study endeavoured to establish the everyday experiences of caregivers whose children with ASD were receiving occupational therapy at Kenyatta National Hospital. The participants reported on the challenges faced, as well as their support needs. Spiritual and cultural beliefs about the causes of ASD strongly influence the experiences of caregivers of children with ASD. Mothers mostly experienced rejection from partners, family and the community, as they were held responsible for the child’s condition. Such negative attitudes towards disability hamper the development of care pathways for children with ASD and their families in Kenya.

## Appendix 1: Focus group question guide

### Focused group discussion guide: Caregivers of children with autism spectrum disorder

Selection criteria: Caregivers of children with autism spectrum disorder for at least one year prior to the study, aged 18 years and above, proficient in Kiswahili

#### Instructions

Copies of the information sheet, informed consent and confidentiality forms should be provided to each participant and read out aloud for the benefit of those who cannot read. Participants should be afforded an opportunity to ask questions and seek clarifications. Verbal agreement should be tape-recorded.

While using the guide, try to ask the questions in the order given while trying to maintain the flow of the discussion. Try to encourage all the group members to participate in the discussion.

#### Introduction

Thank you very much for agreeing to participate in the discussion. Before we start, I would like to remind the participants that there are no right or wrong answers in this discussion. Our interest is to understand what each of you think. Please feel free to be frank and share your point of view, regardless of whether you agree or disagree with what you hear. It is very important that we hear all your views.

You probably prefer that what you say during discussion should not be told to people outside of this group. Please treat the other participants as you would like to be treated by not telling anyone about what you hear in this discussion today.

Let us start by having each person introduced themselves (besides the participants, the members of the research team should introduce themselves and their roles).

Q1. What do you think about the subject that has brought us here today (autism spectrum disorder)?

Q2. Is autism spectrum disorder common in the community where you come from?

Q3. How did you feel when you were told that your child has autism spectrum disorder?

Q4. In your community, who takes care of a child with autism spectrum disorder?

Q5. How has it been like raising a child with autism spectrum disorder?

Q6. How has having a child with autism spectrum disorder affected your relationship with:

- your other children
- your spouse
- your relatives
- your neighbours
- your employer?

Q7. What challenges have you faced in taking care of a child with autism spectrum disorder? (*Probe: Financial challenges? Accessing health care services? Accessing education services? Social challenges?)*

Q8. How do you cope with the challenges identified above?

Q9. Do you receive any kind of support from:

- the government
- religious organisations
- nongovernmental organisations
- local groups (ask to specify)?

Q10. Is there anything that can be done to improve the experience of taking care of children with autism spectrum disorder? (*Probe: by the government? by religious organisations? by schools? by hospitals? by any other group?)*

Q11. Let us summarise the key points from the discussion. Is there anything else that anyone of you would like to add?

Thank you very much for taking your time to take part in this discussion.

## References

[CIT0001] Ambikile J.S. & Outwater A., 2012, ‘Challenges of caring for children with mental disorders: Experiences and views of caregivers attending the outpatient clinic at Muhimbili National Hospital, Dar es Salaam – Tanzania’, *Child and Adolescent Psychiatry and Mental Health* 6(16), 1–11. 10.1186/1753-2000-6-16 22559084 PMC3390284

[CIT0002] American Psychiatric Association, 2013, *Diagnostic and statistical manual*, 5th edn., American Psychiatric Association, Washington, DC, United States.

[CIT0003] Baines D., 2014, *(Un)Learning disability: Recognizing and changing restrictive views of student ability*, Teachers College Press, New York.

[CIT0004] Bayat M., 2014, ‘Understanding views of disability in the Cote d’Ivoire’, Disability & Society 29(1), 30–43. https://doi:10.1080/09687599.2013.768954P8.

[CIT0005] Betancur C., 2011, ‘Etiological heterogeneity in autism spectrum disorders: More than 100 genetic and genomic disorders and still counting’, *Brain Research* 1380, 42–77. 10.1016/j.brainres.2010.11.078 21129364

[CIT0006] Bilgin H. & Kucuk L., 2010, ‘Raising an autistic child: Perspectives from Turkish mothers’, *Journal of Child & Adolescent Psychiatric Nursing* 23(2), 92–99. 10.1111/j.1744-6171.2010.00228.x 20500625

[CIT0007] Brien M.O., 2007, ‘Ambiguous loss in families of children with Autism Spectrum Disorders’, *Family Relationship* 56, 135–146. 10.1111/j.1741-3729.2007.00447.x

[CIT0008] Case-Smith J. & Arbesman M., 2008, ‘Evidence-based review of interventions for autism used in or of relevance to occupational therapy’, *American Journal of Occupational Therapy* 62(4), 416–429. 10.5014/ajot.62.4.416 18712004

[CIT0009] Carpenter C. & Suto M., 2008, *Qualitative research for occupational and physical therapists: A practical guide*, Wiley-Blackwell Publishers, New Jersey, United States.

[CIT0010] Dabrowska A. & Pisula E., 2010, ‘Parenting stress and coping styles in mothers and fathers of pre-school children with autism and Down syndrome’, *Journal of Intellectual Disability Research* 54(3), 266–280. 10.1111/j.1365-2788.2010.01258.x 20146741

[CIT0011] Davidson J. & Orsini M. (eds.), 2013, *Worlds of autism: Across the spectrum of neurological difference*, University of Minnesota Press, Minneapolis, MN.

[CIT0012] Diken I.H., 2006, ‘Turkish mothers’ interpretation of the disability of their children with mental retardation’, *International Journal of Special Education* 21(2), 8–17.

[CIT0013] Ganz M.L., 2007, ‘The lifetime distribution of the incremental societal costs of autism’, *Archives of Paediatrics & Adolescent Medicine* 161(4), 343–349. 10.1001/archpedi.161.4.343 17404130

[CIT0014] Ghanizadeh A. & Alishahi M.J.A., 2009, ‘Helping families caring for children with Autistic Spectrum Disorders’, *Archives of Iranian Medicine* 12(5), 478–482. 19722770

[CIT0015] Gona J., 2016, ‘Autism and other neurodevelopmental disabilities on the Kenyan coast: Challenges, perceived causes and rehabilitation options’, S.I., viewed 07 June 2017, from https://pure.uvt.nl/ws/files/9988857/Gona_Autism_19_01_2016.pdf

[CIT0016] Gona J.K., Newton C.R., Rimba K., Mapenzi R., Kihara M., Van de Vijver F.R. et al., 2015, ‘Parents’ and professionals’ perceptions on causes and treatment options for Autism Spectrum Disorders (ASD) in a multicultural context on the Kenyan coast’, *PLoS One* 10(8), 1–13. 10.1371/journal.pone.0132729 PMC4534101 26267668

[CIT0017] Jaarsma P., 2012, ‘Autism as a natural human variation: Reflections on the claims of the neurodiversity movement’, *Health Care Analysis* 20(1), 1065–3058. 10.1007/s10728-011-0169-9 21311979

[CIT0018] Jolly D., 2011, ‘Ubuntu- a person is a person through other persons’, Master’s degree, Southern Utah University, viewed 31 May 2018, from https://www.suu.edu/hss/comm/masters/capstone/thesis/jolley-d.pdf

[CIT0019] Kenya National Bureau of Statistics, 2015, *Kenya facts and figures, 2015,* viewed n.d., from https://www.knbs.or.ke/download/kenya-facts-2015/

[CIT0020] Kenya Occupational Therapists Association n.d., *KOTA Background,* viewed n.d., from http://www.kotakenya.org/index.php/about/kota-background

[CIT0021] Krefting L., 1991, ‘Rigor in qualitative research: The assessment of trustworthiness’, *American Journal of Occupational Therapy* 45(3), 214–222. 10.5014/ajot.45.3.214 2031523

[CIT0022] Kuhaneck H.M. & Watling R., 2015, ‘Occupational therapy: Meeting the needs of families of people with autism spectrum disorder’, *American Journal of Occupational Therapy* 69(5), 6905170010p1–6905170010p5. 10.5014/ajot.2015.019562 26356652

[CIT0023] Landrigan P.J., 2010, ‘What causes autism? Exploring the environmental contribution’, *Current Opinion Paediatric* 22(2), 219–225. 10.1097/MOP.0b013e328336eb9a 20087185

[CIT0024] Liamputtong P., 2007, *Researching the vulnerable: A guide to sensitive research methods*, Sage Publisher, London.

[CIT0025] Luong J., Yoder M.K. & Canham D., 2009, ‘Southeast Asian parents raising a child with autism: A qualitative investigation of coping styles’, *Journal of School Nursing* 25(3), 222–229. 10.1177/1059840509334365 19364878

[CIT0026] Luther E.H., Canham D.L. & Cureton V.Y., 2005, ‘Coping and social support for parents of children with autism’, *Journal of School Nursing* 21(1), 40–47. 10.1177/10598405050210010901 15660493

[CIT0027] Mak W.W. & Kwok Y.T., 2010, ‘Internalization of stigma for parents of children with autism spectrum disorder in Hong Kong’, *Social Science & Medicine Journal* 70(12), 2045–2051. 10.1016/j.socscimed.2010.02.023 20362379

[CIT0028] Matson J.L. & Kozlowski A.M, 2011, ‘The increasing prevalence of autism spectrum disorders’, *Research in Autism Spectrum Disorders* 5(1), 418–425. 10.1016/j.rasd.2010.06.004

[CIT0029] Njogu K., 2009, ‘Media and disability in Kenya’, *Disability Studies Quarterly* 29(4). 10.18061/dsq.v29i4.983

[CIT0030] Onwuegbuzie A.J., Dickinson W.B., Leech N.L. & Zoran A.G., 2009, ‘A qualitative framework for collecting and analyzing’, *International Journal of Qualitative Methods* 8(3), 1–21. 10.1177/160940690900800301

[CIT0031] Plumb J.C., 2011, ‘The impact of social support and family resilience on parental stress in families with a child diagnosed with an Autism Spectrum Disorder’, unpublished dissertation.

[CIT0032] Republic of Kenya, 2009, *The National Special Needs Education Policy Framework – Final Draft*, Ministry of Education, Nairobi, Kenya.

[CIT0033] Republic of Kenya, 2014, *Kenya Health Policy 2014–2030 – Towards attaining highest standards of health*, Ministry of Health, Afaya House Cathedral Road, Nairobi.

[CIT0034] Ritchie J., Lewis J., Nicholls C.M. & Ormston R. (eds.), 2013, *Qualitative research practice: A guide for social science students and researchers*, Sage, London.

[CIT0035] Rivers J.W. & Stoneman Z., 2003, ‘Sibling relationships when a child has autism: Marital stress and support coping’, *Journal of Autism and Developmental Disorders* 33, 383–394. 10.1023/A:1025006727395 12959417

[CIT0036] Ronald A. & Hoekstra R.A., 2011, ‘Autism spectrum disorders and autistic traits: A decade of new twin studies’, *American Journal of Medical Genetics Part B: Neuropsychiatric Genetics* 156(3), 255–274. 10.1002/ajmg.b.31159 21438136

[CIT0037] Ruiz-Robledillo N., De Andrés-García S., Pérez-Blasco J., González-Bono E. & Moya-Albiol L., 2014, ‘Highly resilient coping entails better perceived health, high social support and low morning cortisol levels in parents of children with autism spectrum disorder’, *Research in Developmental Disability* 35(3), 686–695. 10.1016/j.ridd.2013.12.007 24405793

[CIT0038] Samadi S.A. & McConkey R., 2011, ‘Autism in developing countries: Lessons from Iran’, *Autism Research and Treatment* 2011, 1–11. 10.1155/2011/145359 PMC3420542 22937242

[CIT0039] Seif E.A., Habib D., Noufal A., Farrag S., Bazaid K., Al-Sharbati M. et al., 2008, ‘Use of M-CHAT for a multinational screening of young children with autism in the Arab countries’, *International Review of Psychiatry* 20(3), 281–289. 10.1080/09540260801990324 18569180

[CIT0040] Siman-Tov A. & Kaniel S., 2011, ‘Stress and personal resource as predictors of the adjustment of parents to autistic children: A multivariate model’, *Journal of Autism and Developmental Disorders* 41, 879–890. 10.1007/s10803-010-1112-x 20872059

[CIT0041] Starr E.M. & Foy J.B., 2012, ‘In parents’ voices: The education of children with autism spectrum disorders’, *Remedial and Special Education* 33(4), 207–216. 10.1177/0741932510383161

[CIT0042] Wilson D. & Williams V., 2013, ‘Ubuntu: Development and Framework of a Specific Model of Positive Mental Health’, *Psychology Journal* 10(2), 1–21.

[CIT0043] World Health Organization, 2013a, *Comprehensive and coordinated efforts for the management of autism spectrum disorder*, WHO, Geneva.

[CIT0044] World Health Organization, 2013b, *Autism spectrum disorders and other developmental disorders from raising awareness to building capacity*, World Health Organization, Geneva.

[CIT0045] WHO Rehabilitation 2030, 2017, *A call for action plan: The need to scale up rehabilitation*, World Health Organization, Geneva, viewed 30 December 2015, from http://www.who.int/disabilities/care/NeedToScaleUpRehab.pdf

